# Sedation and weaning from mechanical ventilation: time for ‘best practice’ to catch up with new realities?

**DOI:** 10.1186/2049-6958-9-45

**Published:** 2014-08-29

**Authors:** Giorgio Conti, Jean Mantz, Dan Longrois, Peter Tonner

**Affiliations:** 1 Department of Intensive Care and Anesthesiology, Università Cattolica del Sacro Cuore, Rome, Italy; 2 Anesthesiology Department, Beaujon Hospital, AP-HP, Université Paris-Diderot, Paris, France; 3 Département d’Anesthésie Réanimation Chirurgicale, Hôpital Bichat-Claude Bernard, Université Paris-Diderot, Hôpitaux Universitaires Paris Nord Val de Seine, Paris, France; 4 Department of Anesthesiology and Intensive Care Medicine, Emergency Medicine Hospital Links der Weser GmbH, Bremen, Germany

**Keywords:** Intensive care, Mechanical ventilation, Sedation, Weaning

## Abstract

Delivery of sedation in anticipation of weaning of adult patients from prolonged mechanical ventilation is an arena of critical care medicine where opinion-based practice is currently hard to avoid because robust evidence is lacking. We offer some views on this subject, hoping to stimulate debate among colleagues.

## Introduction

Although even brief scrutiny of MEDLINE and ClinicalTrials.gov reveals that vigour and ingenuity are being directed towards evaluating every aspect of intensive care unit (ICU) medicine, it remains the case that a comprehensive base of evidence is not available for every aspect of practice. In the absence of conclusive evidence, well-founded opinions retain a role in providing a framework for clinical reasoning and decision making.

Whether and how to deliver sedation in anticipation of weaning of adult patients from prolonged mechanical ventilation is one arena where such opinion-based medicine is called for as new methods and resources emerge, as the limitations of some established practices are better appreciated and as we wait for a new generation of randomized controlled trials (RCTs) to provide definitive evidence. In this paper we offer some views on this subject, hoping to stimulate debate among colleagues.

Issues for consideration in this context include:

(1) Definition of weaning and the distribution of weaning experience

(2) Aspects of current practice that should be discarded

(3) Criteria for weaning

(4) Influence of the type/quality of sedation on the success/failure of weaning in ICU patients

(5) Weaning in the context of cooperative sedation (a concept unknown when several pivotal RCTs on weaning were performed).

### Defining weaning

Weaning is the liberation of a patient from mechanical ventilatory support. As such it is a process not an outcome and a process that starts when the decision is taken to intubate a patient. Weaning is thus not the same thing as extubation; rather, extubation may be seen as the culmination of the weaning process.

Maintaining patients on the weaning pathway requires a holistic approach that prioritizes the best preservation of respiratory muscles without excessive stress to patients, maintenance of haemodynamic stability, nutrition and electrolyte status, and attention to patient’s motivation and engagement.

A corollary of this definition of weaning is that non-invasive ventilation (NIV) can be an important and valuable part of the weaning process and an adjunct to extubation. This is demonstrably the case in chronic obstructive pulmonary disease (COPD), as shown in several RCTs [1-3]. NIV is often applied in non-COPD cases of hypoxaemic respiratory failure, though as yet without the substantiation of major RCTs [4,5].

#### *The distribution of weaning experience*

The general pattern of weaning described by Boles et al. [6] remains a broadly accurate summary of routine experience. Thus some 70% of mechanically ventilated patients are successfully weaned at the first attempt. Of the remaining 30%, about 25% experience ‘difficult’ weaning (defined as failure the first time but success within 1 week of the first attempt) and 5% experience prolonged weaning (defined as >1 week with repeated attempts).

The confirmation by the Spanish Lung Failure Collaborative Group that ventilator support can be successfully discontinued in two-thirds of ventilated patients after a 2-h spontaneous breathing trial (SBT) is also compatible with this distribution [7].

### Aspects of current practice that should be discarded

Three elements of what might be termed the classic weaning schedule are either unnecessary or redundant and should be discarded.

#### *Predictors of weaning*

None of the claimed predictors of weaning, whether used alone or in combination, are reliably predictive of weaning success and no time or effort should be wasted on monitoring them for that purpose. This lack of reliability is evident for unitary criteria such as vital capacity, maximal inspiratory pressure and minute ventilation [8-10] and for the respiratory frequency to tidal volume (f/VT) ratio [11], which at one time seemed the most promising practical predictive instrument.

#### *The spontaneous awakening trial*

The adoption by ICUs (certainly in Europe) of awake, cooperative sedation as the standard of care means that the spontaneous awakening trial [12] is now largely an anachronism, relevant only to those narrow sectors of the ICU population where deep sedation is still needed (e.g. traumatic brain injury [TBI] or acute respiratory distress syndrome [ARDS] patients or those receiving neuromuscular blockers [NMBs]). Outside those areas, few ICU patients are too ill to be awake with sedation provided proper attention is given to their needs for analgesia.

#### *Early tracheotomy*

Given that some 70% of patients can expect to be weaned successfully at the first attempt, there is no argument for a generalized use of early tracheotomy as a prelude or adjunct to weaning [13,14]. There may be exceptions to that verdict for brain-injured patients or multiple trauma victims.

### Criteria for weaning

The central question in weaning is when to complete the return to unsupported spontaneous breathing. Readiness should be monitored by ‘dynamic assessment’, anchored by a series of key considerations that emphasize trend and stability, rather than by rigid thresholds.

1. Resolution of the underlying cause of acute respiratory failure

2. Haemodynamic stability, defined as no need for vasoactive/inotropic drugs

3. Adequate neurological status, defined as:

Glasgow Coma Scale score >8^a^ or, if sedated, target Richmond Agitation-Sedation Scale score in the range -2 to 0 achieved with minimal sedation

4. Preferably absence of fever (defined as temperature <38°C)^b^

5. Adequate gas exchange, as indicated by a partial pressure of oxygen:fraction of inspired oxygen ratio >200 with a positive end-expiratory pressure of 5 cmH_2_O

6. Partial pressure of carbon dioxide adjusted to bring blood pH into the normal range [7].

### Weaning in the context of cooperative sedation

Current guidelines on weaning rest very considerably on the findings of RCTs performed during the last decade of the 20^th^ century. One of those studies demonstrated that immediate extubation after a successful SBT accelerates weaning and reduces the duration of mechanical ventilation [7]. Two other studies showed that the ability to breathe spontaneously can be adequately tested by a trial with either T-tube or pressure support of 7 cmH_2_O lasting either 30 or 120 min [15]. Two randomized studies found that synchronized intermittent mandatory ventilation is the worst method of weaning in difficult-to-wean patients [7,16].

However, all those studies required patients to be sedative-free before weaning was attempted. That was a reasonable stipulation given the nature of the sedatives and the sedation protocols then prevailing. In view of the current widespread emphasis on cooperative sedation - a concept undeveloped at the time of those trials - and the increased availability of sedatives free from adverse effects on respiration, there is an evident need for new studies analysing the effects of these new sedation paradigms on the weaning process.

Until such studies are completed, the evidence base for the optimized management of weaning is incomplete. However, it is possible to propose a framework of guidance for current practice.

• Most patients who require sedation in the context of weaning are likely to be among the 30% or so of patients not weaned successfully at the first attempt. Ongoing sedation in the context of weaning will thus be confined most likely to a minority of patients.

• ‘Sedation’ should be described and configured as ‘analgo-sedation’. This word highlights the primacy of pain relief for the delivery of patient comfort before, during and after weaning.

• Minimum sedation is the prerequisite for weaning and should be regarded in any case as the default target in the absence of special circumstances such as severe ARDS, TBI or use of NMBs.

• Use of longer-acting sedatives should be avoided. There should be, in particular, a strong presumption against the use of benzodiazepines, which ought now to be regarded as drugs of last resort in most situations. This latter conclusion is aligned with and draws support from the recent retrospective analyses of Fraser et al. [17].

• Rapid or abrupt termination of an established sedative regimen should be avoided, especially when longer-acting sedatives have been used.

• Step-down sedation, for example substituting propofol for a benzodiazepine, may be needed for patients who have been maintained on longer-acting agents.

• Monitoring of minimally sedated awake, cooperative patients for pain, agitation and delirium (both hyper- and hypo-active) should be systematically conducted at least once a day as part of the weaning routine. (This need not take very long.) When detected, the first response must be to correct the primary cause, not to obscure it with sedation. The general principles and practical guidance enumerated by Barr and colleagues [18] provide the basis for current best practice in this area, but it should be noted that many of those recommendations are based on limited evidence, especially in the area of ICU delirium.

• Identification and effective treatment of pain is a priority, including pain as the cause of agitation. Delirium (in whatever form) is often associated with pain or agitation and in these cases it will likely respond to measures to relieve pain and agitation. We concur with Barr et al. [18] in regarding the Confusion Assessment Method for The Intensive Care Unit and the Intensive Care Delirium Screening Checklist as currently the best instruments for identification of delirium, combining practicability with reliability and accuracy (see also Neto et al. [19]). (It is again important to note that at the time when some major RCTs on weaning were performed, even the concept of ‘delirium’ was poorly described and not incorporated in the decision analysis.)

• The long-term impact of delirium on the trajectory of an ICU patient remains a matter of debate, but a growing body of evidence supports the assertion that delirium is associated with impaired post-ICU cognitive function [20]. There is more certainty about its adverse impact on the patient’s course in the ICU and in particular on the duration of mechanical ventilation. Attention to delirium is thus an essential feature of preparations for weaning, including all aspects of sedation practice. Barr et al. [18] identify continuous infusion of dexmedetomidine as the only pharmacological measure likely to reduce the duration of delirium.

• Because many patients who need sedation as an adjunct to weaning will have experienced prolonged or difficult weaning, effects of sedatives on respiratory function are an important consideration.

• Sedatives that act via γ-aminobutyric acid (GABA)-ergic pathways (e.g., benzodiazepines, opioids and propofol) adversely affect respiratory drive and/or timing. This aspect is very important, as sedation with GABA-ergic and/or morphine-like agents can represent *per se* a source of patient–ventilator asynchrony, prolonging the need for ventilatory support [21].

• Alpha_2_-agonists do not adversely affect respiratory drive and/or timing.

A range of sedative regimens are in use in Europe, reflecting differing circumstances, priorities or perspectives at national or sub-national levels. It is, therefore, infeasible to recommend a single agent or regimen, but for patients undergoing difficult or prolonged weaning there should be a presumption in favour of short-acting sedatives delivered as a continuous infusion. Remifentanil and dexmedetomidine are particularly well suited to that requirement and can be relied on to deliver comfort to patients emerging into spontaneous breathing while also meeting the goal of minimal/awake sedation. Low-dose morphine in the manner advocated by Strøm et al. [22] may also be considered, though there is a widespread assumption (not yet formally tested) that this approach requires a very low (probably 1:1) staff:patient ratio to enable it to be implemented consistently successfully.

Remifentanil should be used as a very low-dose continuous infusion (e.g. 0.05 μg/kg/min) to avoid possible adverse effects on respiration [23].

Dexmedetomidine appears to be particularly well suited to the needs of patients receiving NIV as part of a step-down ventilation strategy or for the prevention (but not treatment) of post-extubation weaning failure. One rationale for this application is that NIV enables some patients to be extubated sooner than might be possible if they were being restored directly from mechanically supported to fully unsupported breathing. Patients proceeding to NIV may, therefore, be extubated sooner and/or at a deeper level of sedation than if they were being restored to unsupported breathing. Dexmedetomidine-based sedation during NIV may, therefore, be used as a step-down measure replacing longer-acting sedatives and to prepare the patient for the final phase of weaning by establishing conscious sedation, just as NIV itself is used as a step-down ventilation measure prior to extubation.

Epstein [24] and Burns and colleagues [25] have noted that the evidence from randomized trials of the benefit of NIV as an aid to weaning or to prevent post-extubation failure is substantially limited to acute-on-chronic respiratory failure, most obviously COPD. Whether or not using NIV under present conditions reflects this limitation and whether an emphasis on newer sedatives such as dexmedetomidine might enhance the success of NIV in this context or expand the range of valid clinical situations in which NIV is advantageous are matters deserving of early investigation.

Other situations in which dexmedetomidine is strongly to be favoured include:

• Patients at risk for weaning syndrome and/or delirium, or otherwise vulnerable to mental deterioration for non-organic reasons

• Patients experiencing sleep deprivation. (Dosages may be adjusted throughout the 24-h cycle to help restore a more normal sleep–wake pattern, in the wider context of measures to promote/protect sleep. Though *inter alia* see Tamrat et al. [26] for a recent systematic review of non-pharmacological measures to promote sleep. The broad conclusion of that work is that there is a lack of good-quality evidence to guide practice in this arena.)

## Conclusions

The time has probably come to revisit all aspects of weaning and weaning protocols in view of recent transformation in sedative practice for critically ill patients [18]. Considering the interrelation between sedation and weaning it is increasingly difficult to justify persisting with practice based on experience and conclusions derived from sedation studies performed with drugs that possess important pharmacological drawbacks and which increasingly are regarded as seldom appropriate except as agents of last resort in an era when little if any use was made of sedation scales and when the significance (or even existence) of delirium was greatly under-appreciated and formed no part of clinical reasoning or procedure.

It is time that the evidence base for sedation and weaning was enlarged to reflect the new realities already in force in many countries. In the short term there should be a concerted effort to address the many areas of practice where the authors of the pain, agitation, and delirium (PAD) guidelines [18] could find either no evidence better than grade C or no evidence at all. As a longer-term strategy we propose that research should aim to provide evidential support for all recommendations of the 2013 PAD guidelines to at least level 1B.

## Endnotes

^a^Weaning success rates in TBI and neurocritical patients are not always nil. This implies that the minimum level of consciousness required for successful weaning may be lower than is generally acknowledged; however, these data do not justify accepting worse than the best achievable level of awareness in non-TBI patients. ^b^Temperature >38°C is not an absolute barrier if the fever is of non-infectious origin. In such cases, the delay implied in waiting several days for temperature to fall below 38°C may itself be an outcome worth trying to avoid.

## Competing interests

GC reports honoraria for lectures from Orion Pharma (Italy), Covidien and Sylcomed. The Catholic University of Rome has received an institutional research grant from Orion Pharma.

JM reports honoraria from Orion Pharma as a guest lecturer at international symposia in intensive care.

DL reports honoraria from Orion Pharma for guest lectures at international symposia in anaesthesia and intensive care, and editorship of *intensetimes*, an electronic journal supported by Orion Pharma.

PT reports honoraria for lectures at national and international symposia from Orion Pharma, Abbvie, Baxter, Bard, B. Braun and Ratiopharm.

## Authors’ contributions

All authors contributed directly to the discussions that gave rise to this article. All authors read and approved the final manuscript.

## References

[B1] Nava S Ambrosino N Clini E Prato M Orlando G Vitacca M Brigada P Fracchia C Rubini F Noninvasive mechanical ventilation in the weaning of patients with respiratory failure due to chronic obstructive pulmonary disease. A randomized, controlled trial Ann Intern Med 1998 128 721 728 10.7326/0003-4819-128-9-199805010-00004 9556465

[B2] Girault C Daudenthun I Chevron V Tamion F Leroy J Bonmarchand G Noninvasive ventilation as a systematic extubation and weaning technique in acute-on-chronic respiratory failure: a prospective, randomized controlled study Am J Respir Crit Care Med 1999 160 86 92 10.1164/ajrccm.160.1.9802120 10390384

[B3] Ferrer M Esquinas A Arancibia F Bauer TT Gonzalez G Carrillo A Rodriguez-Roisin R Torres A Noninvasive ventilation during persistent weaning failure: a randomized controlled trial Am J Respir Crit Care Med 2003 168 70 76 10.1164/rccm.200209-1074OC 12689847

[B4] Vaschetto R Turucz E Dellapiazza F Guido S Colombo D Cammarota G Della Corte F Antonelli M Navalesi P Noninvasive ventilation after early extubation in patients recovering from hypoxemic acute respiratory failure: a single-centre feasibility study Intensive Care Med 2012 38 1599 1606 10.1007/s00134-012-2652-7 22825283

[B5] Burns KEA Meade MO Premji AC Adhikari NKJ Noninvasive ventilation as a weaning strategy for mechanical ventilation in adults with respiratory failure: a Cochrane systematic review CMAJ 2014 186 E112 E122 10.1503/cmaj.130974 24324020 PMC3928231

[B6] Boles JM Bion J Connors A Herridge M Marsh B Melot C Pearl R Silverman H Stanchina M Vieillard-Baron A Welte T Weaning from mechanical ventilation Eur Respir J 2007 29 1033 1056 10.1183/09031936.00010206 17470624

[B7] Esteban A Frutos F Tobin MJ Alía I Solsona JF Valverdú I Fernández R de la Cal MA Benito S Tomás R A comparison of four methods of weaning patients from mechanical ventilation. Spanish Lung Failure Collaborative Group N Engl J Med 1995 332 345 350 10.1056/NEJM199502093320601 7823995

[B8] Gandía F Blanco J Evaluation of indexes predicting the outcome of ventilator weaning and value of adding supplemental inspiratory load Intensive Care Med 1992 18 327 333 10.1007/BF01694360 1469159

[B9] Sassoon CSH Mahutte CK Airway occlusion pressure and breathing pattern as predictors of weaning outcome Am Rev Respir Dis 1992 148 860 866 8214939 10.1164/ajrccm/148.4_Pt_1.860

[B10] Yang K Tobin MJ A prospective study of indexes predicting outcome of trials of weaning from mechanical ventilation N Engl J Med 1991 24 1445 1450 2023603 10.1056/NEJM199105233242101

[B11] Conti G Montini L Pennisi MA Cavaliere F Arcangeli A Bocci MG Proietti R Antonelli M A prospective, blinded evaluation of indexes proposed to predict weaning from mechanical ventilation Intensive Care Med 2004 30 830 836 10.1007/s00134-004-2230-8 15127195

[B12] Kress JP Pohlman AS O’Connor MF Hall JB Daily interruption of sedative infusions in critically ill patients undergoing mechanical ventilation N Engl J Med 2000 342 1471 1477 10.1056/NEJM200005183422002 10816184

[B13] Young D Harrison DA Cuthbertson BH Rowan K TracMan Collaborators Effect of early vs late tracheostomy placement on survival in patients receiving mechanical ventilation: the TracMan randomized trial JAMA 2013 309 2121 2129 10.1001/jama.2013.5154 23695482

[B14] Terragni PP Antonelli M Fumagalli R Faggiano C Berardino M Pallavicini FB Miletto A Mangione S Sinardi AU Pastorelli M Vivaldi N Pasetto A Della Rocca G Urbino R Filippini C Pagano E Evangelista A Ciccone G Mascia L Ranieri VM Early vs late tracheotomy for prevention of pneumonia in mechanically ventilated dult ICU patients: a randomizedcontrolled trial JAMA 2010 303 1483 1489 10.1001/jama.2010.447 20407057

[B15] Esteban A Alía I Gordo F Fernández R Solsona JF Vallverdú I Macías S Allegue JM Blanco J Carriedo D León M de la Cal MA Taboada F Gonzalez De Velasco J Palazón E Carrizosa F Tomás R Suarez J Goldwasser RS Extubation out come after spontaneous breathing trials with T-tube or pressure support ventilation. Spanish Lung Failure Collaborative Group Am J Respir Crit Care Med 1997 156 459 465 10.1164/ajrccm.156.2.9610109 9279224

[B16] Brochard L Rauss A Benito S Conti G Mancebo J Rekik N Gasparetto A Lemaire F Comparison of three methods of gradual withdrawal from ventilatory support during weaning from mechanical ventilation Am J Respir Crit Care Med 1994 150 896 903 10.1164/ajrccm.150.4.7921460 7921460

[B17] Fraser GL Devlin JW Worby CP Alhazzani W Barr J Dasta JF Kress JP Davidson JE Spencer FA Benzodiazepine versus nonbenzodiazepine-based sedation for mechanically ventilated, critically ill adults: a systematic review and meta-analysis of randomized trials Crit Care Med 2013 41 9 Suppl 1 S30 S38 23989093 10.1097/CCM.0b013e3182a16898

[B18] Barr J Fraser GL Puntillo K Ely EW Gélinas C Dasta JF Davidson JE Devlin JW Kress JP Joffe AM Coursin DB Herr DL Tung A Robinson BR Fontaine DK Ramsay MA Riker RR Sessler CN Pun B Skrobik Y Jaeschke R American College of Critical Care Medicine Clinical practice guidelines for the management of pain, agitation, and delirium in adult patients in the intensive care unit Crit Care Med 2013 41 263 306 23269131 10.1097/CCM.0b013e3182783b72

[B19] Neto AS Nassar AP Jr Cardoso SO Manetta JA Pereira VG Espósito DC Damasceno MC Slooter AJ Delirium screening in critically ill patients: a systematic review and meta-analysis Crit Care Med 2012 40 1946 1951 10.1097/CCM.0b013e31824e16c9 22610196

[B20] Pandharipande PP Girard TD Jackson JC Morandi A Thompson JL Pun BT Brummel NE Hughes CG Vasilevskis EE Shintani AK Moons KG Geevarghese SK Canonico A Hopkins RO Bernard GR Dittus RS Ely EW BRAIN-ICU Study Investigators Long-term cognitive impairment after critical illness N Engl J Med 2013 369 1306 1316 10.1056/NEJMoa1301372 24088092 PMC3922401

[B21] Vaschetto R Cammarota G Colombo D Longhini F Grossi F Giovanniello A Della Corte F Navalesi P Effects of propofol on patient-ventilator synchrony and interaction during pressure support ventilation and neurally adjusted ventilatory assist Crit Care Med 2014 42 74 82 10.1097/CCM.0b013e31829e53dc 23982026

[B22] Strøm T Martinussen T Toft P A protocol of no sedation for critically ill patients receiving mechanical ventilation: a randomised trial Lancet 2010 375 475 480 10.1016/S0140-6736(09)62072-9 20116842

[B23] Cavaliere F Antonelli M Arcangeli A Conti G Costa R Pennisi MA Proietti R A low-dose remifentanil infusion is well tolerated for sedation in mechanically ventilated, critically-ill patients Can J Anaesth 2002 49 1088 1094 10.1007/BF03017909 12477685

[B24] Epstein SK Noninvasive ventilation to shorten the duration of mechanical ventilation Respir Care 2009 54 198 208 19173752

[B25] Burns KE Adhikari NK Keenan SP Meade MO Noninvasive positive pressure ventilation as a weaning strategy for intubated adults with respiratory failure Cochrane Database Syst Rev 2010 8 CD004127 20687075 10.1002/14651858.CD004127.pub2

[B26] Tamrat R Huynh-Le MP Goyal M Non-pharmacologic interventions to improve the sleep of hospitalized patients: A systematic review J Gen Intern Med 2014 29 788 795 24113807 10.1007/s11606-013-2640-9 PMC4000341

